# Multiple Abdominal Cocoons: An Unusual Presentation of Intestinal Obstruction and a Diagnostic Dilemma

**DOI:** 10.1155/2015/282368

**Published:** 2015-03-29

**Authors:** Mohammad Zain Sohail, Shumaila Hasan, Benan Dala-Ali, Shahanoor Ali, M. A. Hashmi

**Affiliations:** ^1^Department of Trauma & Orthopaedics, Whipps Cross University Hospital, Whipps Cross Road, Leytonstone, London E11 1NR, UK; ^2^Department of Neurosurgery, The Royal London Hospital, Whitechapel Road, Whitechapel, London E1 1BB, UK; ^3^Department of General Surgery, Kettering General Hospital, Rothwell Road, Kettering, Northamptonshire NN16 8UZ, UK; ^4^Department of General Surgery, Shifa International Hospitals Ltd., Pitras Bukhari Road, No. H-8/4, Islamabad, Pakistan

## Abstract

Sclerosing encapsulating peritonitis (SEP) or abdominal cocoon is a rare acquired condition with an unknown aetiology. It is characterized by encapsulation of the small bowel by a fibrous membrane and can lead to intestinal obstruction. We present the case of a 42-year-old gentleman with a history of hepatitis C, tuberculosis, and previous abdominal surgery, who presented with subacute intestinal obstruction. Surgical exploration of the abdomen revealed that the entire contents were enclosed into three distinct sacs by a dense fibrous membrane. Excision of the sacs was performed followed by adhesiolysis. This is believed to be the first reported case of multiple cocoons within the abdominal cavity. The case is discussed with reference to the literature.

## 1. Introduction

Abdominal cocoon is a rare entity and a clinical curiosity in the surgical field. It is characterized by encapsulation of part or whole of the small bowel by a fibrous membrane [1] and can present with intestinal obstruction [2]. The primary form is idiopathic and more common, and secondary form is associated with a myriad of other conditions, including liver cirrhosis, abdominal tuberculosis, and previous abdominal surgery. Surgical treatment including excision of the fibrous membranes and adhesiolysis remains the treatment of choice.

We present the case of a middle-aged male with a history of hepatitis C, tuberculosis (TB), and previous abdominal surgery, presenting with subacute intestinal obstruction secondary to abdominal cocoon. This is believed to be the first reported case of multiple cocoons in the abdomen and one of a few reported cases of abdominal cocoon in association with hepatitis C [3, 4].

## 2. Case Report

A 42-year-old man presented to the surgical assessment unit with a five-day history of abdominal distention and vomiting and a two-month history of colicky and intermittent abdominal pain in the periumbilical region. He had no specific exacerbating factors for the pain, and he found some degree of relief by taking paracetamol and antispasmodics. He had been experiencing a profound decrease in appetite and had lost over 7 kilograms of weight over the previous few months. No other significant constitutional symptoms of fever or night sweats were positive in the history.

The patient had a significant past medical history of tuberculous monoarthritis of the left knee which was diagnosed 9 years prior to presentation. He was commenced on antituberculous therapy (ATT), which he prematurely stopped after four months of treatment without seeking medical advice due to abdominal discomfort. The patient also had a past history of acid peptic disease which was complicated by duodenal perforation for which he had a laparotomy over 18 years prior to the presentation. The patient required a blood transfusion but otherwise had a good postoperative recovery from the operation. When questioned further about this, he and his family were unable to provide further information. The social history confirmed that he was a smoker with a 20-pack year history of smoking. There was no history of alcohol use, and the patient was a taxi driver by profession.

On clinical examination, he had a cachectic appearance and was apyrexial and haemodynamically stable. On palpation of the abdomen, there was a mass in the umbilical region measuring 5 × 6 cm that was firm, nontender, and immobile and had indistinct margins. There was evidence of ascites. Bowel sounds were present and normal. No lymphadenopathy was found on physical examination and digital rectal examination was essentially unremarkable.

Blood investigations initially found microcytic hypochromic anaemia (haemoglobin of 10.4 with MCV of 72). Leukocyte count was 8.4, C-reactive protein (CRP) count was 22, and he had an erythrocyte sedimentation rate (ESR) of 55. The liver function tests were mildly deranged with a raised alanine aminotransferase of 54, aspartate aminotransferase of 66, and a normal bilirubin of 1.0. The coagulation screen showed an international normalized ratio (INR) of 1.4, and the hepatitis serology was positive for antihepatitis C antibodies.

An ultrasound of the abdomen showed multiple well defined masses with matted gut loops and the presence of ascites. An ultrasound guided paracentesis was performed under local anaesthetic and revealed a turbid purulent fluid, which was sent for microbiology, cytology,* Mycobacterium tuberculosis* (MTB) DNA polymerase chain reaction (PCR), and acid fast bacillus (AFB) cultures. The fluid was negative for any organism on grams stain and had a high protein and white cell count. Cytology was negative for atypical cells. Whilst awaiting the results of the AFB cultures and MTB DNA by PCR, a provisional diagnosis of tuberculosis was made after discussion with the infectious diseases department. The patient was commenced on first line ATT, comprising isoniazid (INH), rifampicin (RMP), pyrazinamide (PZA), and ethambutol (EMB). He improved with conservative management and was discharged three days after admission.

The patient did not attend his follow-up appointment. However, it was later discovered that both cultures and MTB DNA by PCR was negative in the ascitic fluid.

The patient presented four months later to the emergency department with severe abdominal pain and vomiting for two days. On clinical examination, he was apyrexial and haemodynamically stable. Abdominal examination revealed generalized abdominal tenderness with no rigidity or guarding, mild splenomegaly, and sluggish bowel sounds. A repeat ultrasound demonstrated matted gut loops, coarse hepatic echo texture, moderate ascites, and splenomegaly. This raised the suspicion of chronic liver disease (CLD). A computerized tomography (CT) of the abdomen revealed a 9 × 5 cm mass in the right iliac fossa, matted gut loops, enlarged lymph nodes, and splenomegaly. The patient did not improve despite conservative management and an explorative laparotomy was performed five days after admission.

**Figure 1:**
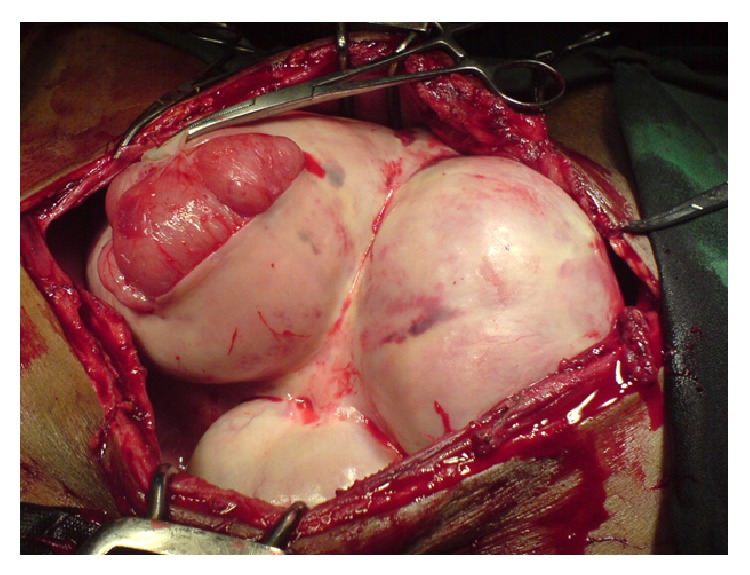
Three large cocoons enclosing the whole of the abdominal contents.

**Figure 2:**
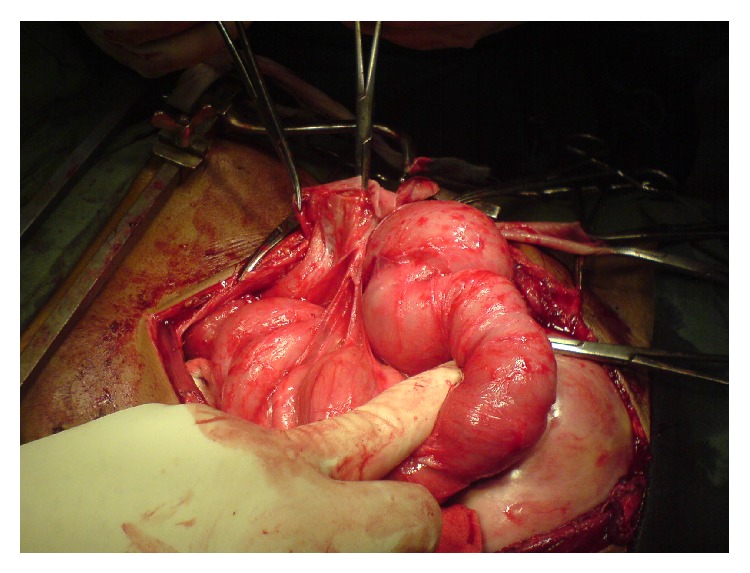
Densely adherent gut loops.

**Figure 3:**
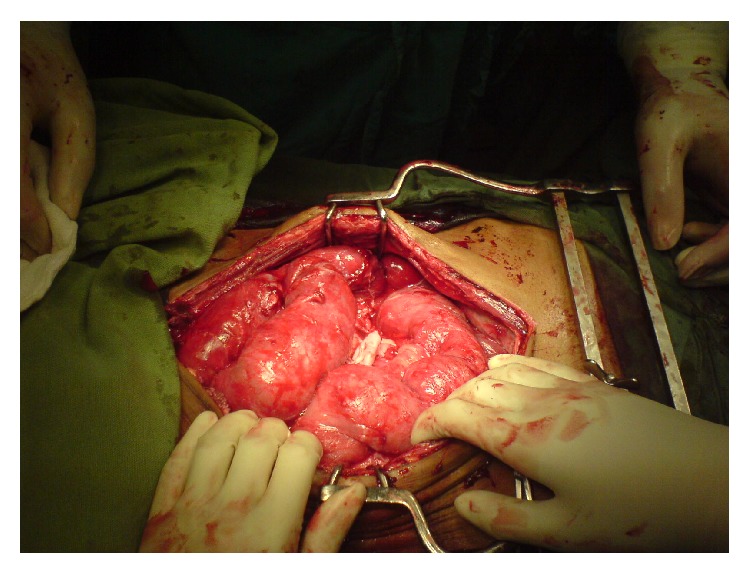
The abdominal contents after adhesiolysis.

Peroperatively, it was found that a dense membrane had enclosed the whole of the abdominal contents against the posterior abdominal wall to form three cocoons (Figure 1). A large cocoon was found extending from the left hypochondrium to the epigastric region (cocoon 1), a second large cocoon was extending from the epigastric region to the pelvis (cocoon 2), and a third smaller cocoon was in the right iliac fossa (cocoon 3). There was also a moderate amount of purulent ascites found within the abdomen. The cocoons were dissected and the membrane was carefully excised to reveal densely adherent loops of gut (Figure 2). Cocoon 1 was found to contain the spleen, a segment of the transverse bowel, small bowel, stomach, and greater omentum. Cocoon 2 also contained portions of the small bowel tightly matted against each other. Cocoon 3 contained the caecum and terminal ileum. The cocoons were separated by thick sheaths of fibrous tissue adherent to the posterior abdominal wall. The encasing membrane was sent for immediate histological analysis as a frozen section and samples of the membrane and ascites were also sent for routine cytology, histopathology, and microbiology. The frozen section came back negative for malignancy and so an extensive adhesiolysis of the gut was done from the duodenojejunal flexure to the ileocaecal junction and was found to be wholly viable (Figure 3). The abdomen was closed after saline irrigation and placement of drain.

The samples taken during the surgical exploration were later found to be negative for MTB DNA by PCR and the histopathological report of the encasing membrane revealed fibrosis and chronic inflammation, negative for granulomatous inflammation and malignancy.

Postoperatively, the patient was managed in the surgical intensive care unit for inotropic and ventilatory support. Despite optimal management, the patient developed hepatorenal shutdown and coagulopathy. The patient later developed an upper gastrointestinal (GI) bleeding and sepsis. Endoscopic gastroduodenoscopy (EGD) revealed grade III calcified varices but no evidence of fresh blood. He was transfused multiple units of PRBCs, platelets, and fresh frozen plasma (FFP). The patient then developed enterohepatic encephalopathy and died 14 days after his surgery.

## 3. Discussion

Sclerosing encapsulating peritonitis (SEP) is a rare acquired malformation [5], the exact aetiology of which is still unknown [6]. The first documented case was observed by Owtschinnikow in 1907, who labeled it peritonitis chronica fibrosa incapsulata [7], and the term abdominal cocoon was coined by Foo et al. in 1978 [8].

SEP is classically divided into primary and secondary forms. The primary, or idiopathic form, is more common and has been described in adolescent females from tropical and subtropical regions. The literature suggests that it may arise from a subclinical primary viral peritonitis, as an immunological reaction to gynaecological infections or due to retrograde menstruation [8]. However, doubts have arisen over these suggestions as the condition has also been reported in children, postmenopausal females, and males [9–11].

The secondary form of SEP has been reported in association with abdominal tuberculosis [1, 2, 5, 12], liver cirrhosis, peritoneal chemotherapy [13], continuous ambulatory peritoneal dialysis (CAPD), and automated peritoneal dialysis (APD) [14]. It has also been noted in patients who had previous abdominal surgery, *β*-blocker practolol intake, liver transplant, sarcoidosis, systemic lupus erythematosus (SLE), gastrointestinal malignancy [15], recurrent peritonitis, and ventriculoperitoneal and peritoneovenous shunts [12].

In this particular case, there were three possible factors that could have individually, or in coalition, led to the formation of abdominal cocoon, tuberculosis, hepatitis C, and/or previous laparotomy. The tuberculosis seemed less likely as MTB DNA by PCR was repeatedly negative in the ascitic fluid and the histopathology of the encasing membrane was negative for caseating granulomatous inflammation. The previous laparotomy for duodenal perforation was considered as a possible cause as the literature has shown an association between abdominal surgeries and cocoons [15]. Hepatitis C has previously been mentioned twice in literature in association with SEP, both times resulting in poor prognosis [3, 4].

The majority of abdominal cocoon cases reported in the past have been incidental findings during surgery, mainly due to the nonspecific clinical features of SEP and the reduced awareness of the condition [5]. Loss of appetite, malnutrition, and weight loss are common presenting symptoms [16]. Nausea, vomiting, episodes of small bowel obstruction, abdominal pain, and distension are other frequent features at presentation [17]. Imaging studies are now crucial for the diagnosis of SEP. In the past, Barium meal follow-through was the main imaging modality utilized to help diagnose the condition. In 1983, Sieck et al. described the classical barium finding of a configuration of dilated small bowel loops in a fixed U-shaped cluster or a “cauliflower sign” [18]. However, these are often not present and are nonspecific [12]. Contrast-enhanced CT is the most helpful modality, with the characteristic appearances including small bowel segments encapsulated by a dense fibrous membrane [16]. More recently, magnetic resonance (MR) images have been acquired and compared with CT and have shown that MR images where similar, if not better, than CT images [19].

SEP has been successfully treated through both medical and surgical methods. There have been reports of corticosteroid and tamoxifen therapy having success in patients with secondary SEP and fibrosclerotic disease [14, 20], although it should be noted that there have been cases of relapse through this method [2]. Surgery remains the treatment of choice [21]. The excision of the encapsulating membrane with adhesiolysis has led to favorable outcomes [22]. More recently, laparoscopic methods have been employed with excellent results [23, 24].

This case has highlighted the severity of the SEP. It remains potentially fatal, and a review of the literature has revealed that the two previously documented cases of abdominal cocoon in association with hepatitis C related liver disease both resulted in mortality [3, 4].

## 4. Conclusion

SEP is a rare but potentially fatal condition. It is essential that clinicians are aware of the diagnosis and treat it accordingly. Although most cases of abdominal cocoon have a favourable outcome, the addition of comorbidities such as CLD or hepatitis can result in a poorer prognosis. This is the first documented case of multiple cocoons within the abdominal cavity. It is possible that the poor outcome of this case may be attributed to having* multiple* cocoons rather than a single cocoon, as in other reported cases.
